# Supplementary material for: Deletion of wfs1 Impairs Oligodendrocyte Precursor Cells Dorsal Distribution and Myelination Through the wfs1-hmgcs1 Axis in Zebrafish
Source: Biology (Basel). 2026 Aug 16;15(16):1402. doi: 10.3390/biology15161402 (PMC13510151; doi:10.3390/biology15161402)
Supplement: Supplementary file 1 [file biology-15-01402-s001.zip › File S1-Transcriptome sequencing data and differentially expressed genes (DEGs) in wfs1-- zebrafish at 5 dpf/2.KEGG/wfs1KOvsWT/GSEA/WT/BETA_ALANINE_METABOLISM/BETA_ALANINE_METABOLISM(DRE00410).html]

Details for gene set BETA\_ALANINE\_METABOLISM(DRE00410)[GSEA]

|  || Dataset | deg.sample |
| Phenotype | sample.cls |
| Upregulated in class | WT |
| GeneSet | BETA\_ALANINE\_METABOLISM(DRE00410) |
| Enrichment Score (ES) | -0.29059547 |
| Normalized Enrichment Score (NES) | -1.2063531 |
| Nominal p-value | 0.23938224 |
| FDR q-value | 0.47171158 |
| FWER p-Value | 1.0 |
Table: GSEA Results Summary

  

Fig 1: Enrichment plot: BETA\_ALANINE\_METABOLISM(DRE00410)      
 Profile of the Running ES Score & Positions of GeneSet Members on the Rank Ordered List

  

| PROBE | DESCRIPTION (from dataset) | GENE SYMBOL | GENE\_TITLE | RANK IN GENE LIST | RANK METRIC SCORE | RUNNING ES | CORE ENRICHMENT || 1 | 541429 | aoc2 |  |  | 862 | 2.303 | 0.0429 | No |
| 2 | 368239 | aldh2.2 |  |  | 1221 | 1.881 | 0.0888 | No |
| 3 | 553401 | hadhaa |  |  | 1608 | 1.593 | 0.1250 | No |
| 4 | 447920 | aldh3a2b |  |  | 1830 | 1.444 | 0.1619 | No |
| 5 | 492476 | csad |  |  | 2749 | 1.096 | 0.1664 | No |
| 6 | 100144569 | dpys |  |  | 3351 | 0.963 | 0.1768 | No |
| 7 | 445288 | acads |  |  | 3897 | 0.848 | 0.1854 | No |
| 8 | 100000859 | ehhadh |  |  | 4565 | 0.737 | 0.1869 | No |
| 9 | 100005587 | aldh9a1a.1 |  |  | 5134 | 0.663 | 0.1892 | No |
| 10 | 564675 | paox1 |  |  | 5446 | 0.638 | 0.1988 | No |
| 11 | 405829 | dpydb |  |  | 5638 | 0.623 | 0.2117 | No |
| 12 | 100038790 | zgc:163121 |  |  | 5957 | 0.591 | 0.2197 | No |
| 13 | 449662 | acox1 |  |  | 6280 | 0.559 | 0.2265 | No |
| 14 | 399481 | aldh9a1b |  |  | 6718 | 0.518 | 0.2285 | No |
| 15 | 393462 | aldh2.1 |  |  | 8615 | 0.394 | 0.1810 | No |
| 16 | 100000026 | aldh3a1 |  |  | 8825 | 0.379 | 0.1859 | No |
| 17 | 436647 | aldh6a1 |  |  | 9402 | 0.340 | 0.1782 | No |
| 18 | 322660\_1 | upb1 |  |  | 9992 | 0.304 | 0.1689 | No |
| 19 | 793834 | hadhab |  |  | 10077 | 0.298 | 0.1753 | No |
| 20 | 100329827 | gad1a |  |  | 10096 | 0.296 | 0.1838 | No |
| 21 | 406421 | acox3 |  |  | 12372 | 0.170 | 0.1175 | No |
| 22 | 492710 | aldh16a1 |  |  | 14167 | 0.084 | 0.0637 | No |
| 23 | 387290 | smox |  |  | 17750 | -0.065 | -0.0468 | No |
| 24 | 368912 | echs1 |  |  | 18310 | -0.089 | -0.0616 | No |
| 25 | 378441 | gad1b |  |  | 19299 | -0.135 | -0.0885 | No |
| 26 | 378968 | abat |  |  | 19919 | -0.162 | -0.1030 | No |
| 27 | 327288 | cndp2 |  |  | 20209 | -0.175 | -0.1068 | No |
| 28 | 282559 | aldh3b1 |  |  | 20742 | -0.200 | -0.1174 | No |
| 29 | 334197 | aldh7a1 |  |  | 21094 | -0.221 | -0.1217 | No |
| 30 | 550403 | gad2 |  |  | 21132 | -0.223 | -0.1161 | No |
| 31 | 100006238 | aldh9a1a.2 |  |  | 23161 | -0.338 | -0.1695 | No |
| 32 | 557008 | aldh3b2 |  |  | 24033 | -0.394 | -0.1849 | No |
| 33 | 798364 | hibch |  |  | 27149 | -0.642 | -0.2632 | No |
| 34 | 323653 | aldh3a2a |  |  | 28024 | -0.745 | -0.2680 | Yes |
| 35 | 100037349 | mlycd |  |  | 28119 | -0.759 | -0.2479 | Yes |
| 36 | 768165 | dpyda.1 |  |  | 28815 | -0.881 | -0.2429 | Yes |
| 37 | 100006238\_1 | aldh9a1a.2 |  |  | 29971 | -1.231 | -0.2418 | Yes |
| 38 | 322660 | upb1 |  |  | 30928 | -1.889 | -0.2145 | Yes |
| 39 | 100005587\_1 | aldh9a1a.1 |  |  | 31308 | -2.447 | -0.1520 | Yes |
| 40 | 100536795 | wu:fb14d09 |  |  | 31372 | -2.574 | -0.0758 | Yes |
| 41 | 541429\_1 | aoc2 |  |  | 31543 | -3.032 | 0.0109 | Yes |
Table: GSEA details [plain text format]

  

Fig 2: BETA\_ALANINE\_METABOLISM(DRE00410)      
 Blue-Pink O' Gram in the Space of the Analyzed GeneSet

  

Fig 3: BETA\_ALANINE\_METABOLISM(DRE00410): Random ES distribution      
 Gene set null distribution of ES for **BETA\_ALANINE\_METABOLISM(DRE00410)**
